# Myelin water fraction mapping with joint inversion of gradient-echo and spin-echo data

**DOI:** 10.1007/s10334-025-01235-5

**Published:** 2025-03-07

**Authors:** Ségolène Dega, Mónica Ferreira, Marten Veldmann, Rüdiger Stirnberg, Hendrik Paasche, Tony Stöcker

**Affiliations:** 1 UFZ - Helmholtz Centre for Environmental Research (UFZ), Leipzig, Germany; 2 German Center for Neurodegenerative Diseases (DZNE), Bonn, Germany; 3 University of Bonn, Bonn, Germany; 4 Department of Physics and Astronomy, University of Bonn, Bonn, Germany

**Keywords:** Myelin water imaging, Myelin water fraction, *T*_2_ and ${T}_{2}^{*}$ relaxation spectra, Joint inversion

## Abstract

**Objective:**

Accurate estimation of brain myelin-water content from multi-echo data is challenging due to the inherent ill-posedness of the inversion problem. In this study, we propose a novel method for myelin-water imaging that jointly utilizes gradient-echo and spin-echo imaging data to enhance the accuracy of myelin-water estimation.

**Material and methods:**

Multi-echo gradient-echo and spin-echo data were simulated and acquired in vivo. The simulations are based on a parameterized myelin and free water signal model, which is also used for the inversion by means of nonlinear local-search optimization. Single inversions of the individual datasets as well as joint inversion of the combined datasets were performed on simulated and real data. While single inversions estimate either the ${T}_{2}$ or ${T}_{2}^{*}$ relaxation spectrum, the joint inversion estimates both spectra simultaneously.

**Results:**

Simulation results show that the accuracy of myelin-water imaging improves when jointly inverting gradient-echo and spin-echo synthetic data. In vivo experiments show that the joint inversion of both datasets leads to sharper and more distinct myelin-water images as compared to the individual inversions.

**Discussion:**

Our method addresses the ill-posed nature of the myelin-water inversion problem by leveraging complementary information from multi-echo gradient-echo and multi-echo spin-echo imaging sequences, thus improving the reliability of myelin-water quantification.

**Supplementary Information:**

The online version contains supplementary material available at 10.1007/s10334-025-01235-5.

## Introduction

Myelin, a lipid-rich substance surrounding neuronal axons, plays a critical role in facilitating rapid signal transmission in the central nervous system. Alterations in myelin content are implicated in various neurologic disorders, making accurate myelin measurements essential for both research and clinical applications. MRI can indirectly assess myelin content by estimating the amount of myelin water (MW), i.e., the amount of water trapped in the myelin sheath [1]. Myelin water has an influence on multiple MRI contrasts, and meanwhile several myelin-water imaging (MWI) methods exist which are either based on ${T}_{1}$, ${T}_{2}$, ${T}_{2}^{*}$, or MT (magnetization transfer) contrast [2, 3]. For instance, MW has shorter transverse relaxation times than the surrounding axonal and extra-cellular water (AEW). Originally, ${T}_{2}$ and ${T}_{2}^{*}$ contrasts were used to estimate the myelin-water fraction ($MWF$ = amount of MW divided by the total amount of water) from the peak areas of transverse relaxation spectra [4, 5]. Here, either multi-echo spin-echo (ME-SE) or multi-echo gradient-echo (ME-GE) experiments can be employed to measure the multi-exponential ${T}_{2}$ or ${T}_{2}^{*}$ decay signals, respectively. Afterward the relaxation spectra are obtained from data fitting with a suitable signal model. However, this process often suffers from limited accuracy and precision, primarily due to the ill-posed nature of the inversion problem. The MR signal from a single imaging voxel is the superposition of many decay signals with different relaxation times (MW and AEW). In case of a simple multi-exponential decay signal model, estimating those components from their sum is mathematically equivalent to the inverse Laplace transform [2]. Since exponential functions are mathematically not orthogonal, this is a highly ill-posed inversion problem and the fitting is very sensitive to noise [3]. In order to get stable fit results regularization is typically required. Adding a regularization term to the objective function improves the conditioning of the minimization problem, thus enabling a direct numerical solution. Commonly Tikhonov regularization is employed, which typically results in smooth relaxation spectra with minimum energy (${L}_{2}$-norm). Regularization enforces a numerical stable solution, however, it also biases the MWF estimates [3]. In addition to the ill-posed nature of the inversion problem, MWF estimation is often further confounded by an underdetermined system of equations: multi-echo signal data are acquired at $N$ different echo times ($TE$) from which a relaxation spectrum with $M>N$ support points shall be estimated [2]. From the infinite number of possible solutions, the inversion chooses the one which minimizes the objective function (including regularization for stabilization). Commonly, this minimization problem is solved with the nonnegative least squares (NNLS) approach, where no assumption is made on the shape of the relaxation spectrum. The approach is fast, easy to apply and has the additional advantage that the result does not depend on a starting model (in contrast to parameterized local-search optimization), which may explain its popularity in MW imaging. However, it has the disadvantage that a large number of unknowns—the entire relaxation distribution has to be fitted, hence resulting in the above-mentioned under-determination. Alternatively, it has been shown that fitting a parameterized relaxation spectrum vastly reduces the number of unknowns and improves fitting stability in case of low SNR ME-SE data. Raj et al. modeled the ${T}_{2}$ relaxation distribution as a sum of Gaussian functions representing the different compartments [6]. Then, the inversion problem was solved with an iterative nonlinear least-squares optimization approach. The approach was also applied to ME-GE data with Dirac function (“Delta-peaks”) [7], where additionally the complex signal was utilized. The ME-GE signal depends on susceptibility-induced frequency shifts due to white matter fiber orientation [8]. Therefore, using additional phase information improves MWF estimates based on ME-GE data [3].

Nevertheless, in all these cases, the ill-posed nature of the inversion problem and the concomitant fitting instabilities remain, typically requiring strong regularization. However, ME-GE and ME-SE provide $MWF$ estimates via partially independent tissue properties: while the ME-SE signal solely depends on proton density, ${T}_{1}$ and ${T}_{2}$, the ME-GE signal in addition depends on $T_{2}^{\prime } = \left( {1/T_{2}^{*} - 1/T_{2} } \right)^{ - 1}$ and the above-mentioned frequency shifts. Similar situations are often met in geophysics, where (partially) independent measures provide estimates of physical parameters, each resulting from a highly ill-posed inversion problem. In order to improve the accuracy and precision of the parameter estimates, joint inversion is a geophysical approach on simultaneously inverting multiple data sets influenced by common subsurface physical parameters [9–11]. In this study, we propose such a joint inversion approach to MWI that capitalizes on the complementary information provided by ME-GE and ME-SE imaging sequences. By jointly inverting data from both sequences using parameterized ${T}_{2}$ and ${T}_{2}^{*}$ relaxation spectra, our method aims to mitigate the ill-posedness of the individual inversion problems and enhance the reliability of MW estimation. We hypothesize that the combination of ME-GE and ME-SE data can provide complementary information sensitive to related but different tissue properties (${T}_{2}$ and ${T}_{2}^{*}$), thus improving the robustness and accuracy of MW quantification. Potentially, this can be used to relax regularization. Through simulations and in vivo experiments we demonstrate the efficacy of the proposed method in improving MWI.

## Theory

### ME-GE and ME-SE signal decay models

Under the assumption that ${T}_{2}$ and ${T}_{2}^{*}$ are constant within the acquisition time window (e.g., not influenced by exchange processes), the MR signals of ME-GE and ME-SE sequences can be expressed as integrals [3]

1 $$S_{GE} \left( t \right) = \mathop \int \limits_{0}^{\infty } \,\Omega_{GE} \left( {T_{2}^{*} } \right)\,\exp \left[ { - t/T_{2}^{*} } \right]\;dT_{2}^{*}$$

2 $$S_{SE} \left( t \right) = \mathop \int \limits_{0}^{\infty } \,\Omega_{SE} \left( {T_{2} } \right)\,S_{EPG} \left( {t;\alpha_{r} ,T_{1} ,T_{2} } \right)\;{\text{d}}T_{2}$$

where ${\Omega }_{SE}$ and ${\Omega }_{GE}$ denote the ${T}_{2}$ and ${T}_{2}^{*}$ relaxation spectra (or distributions) of an imaging voxel. The first equation assumes that the effective transverse relaxation is governed by a simple multi-exponential decay. Thus, the composite ME-GE signal is the forward Laplace transform of the ${T}_{2}^{*}$ distribution. In case of perfect 180° refocusing, the ME-SE signal would follow the same signal model with ${T}_{2}$ instead of ${T}_{2}^{*}$. However, it is typically not possible to obtain perfect refocusing pulses in real experiments due to subject-induced transmit field inhomogeneities. Therefore, it is necessary to account for signal deviations originating from stimulated echoes generated in the CPMG echo train [12]. These signal components can be conveniently modeled with the extended phase graph (EPG) algorithm [13, 14]. Accordingly, ${S}_{EPG}\left(t;{\alpha }_{r},{T}_{1},{T}_{2}\right)$ in Eq. (2) denotes the ME-SE signal at the echo time $t$ computed with the EPG formalism. It depends on the refocusing flip angle, ${\alpha }_{r}$, and the relaxation times ${T}_{1}$ and ${T}_{2}$. However, the ${T}_{1}$ dependence is weak in case of brain tissue with ${T}_{1}\gg {T}_{2}$, and it is sufficient to approximate ${T}_{1}\approx 1$ s with minimal effect on the signal amplitude (and, therefore, subsequent MWF estimation) [15].

Discretization of the integrals in Eqs. (1) and (2) for a finite number of echo times and a grid of relaxation times, ${T}_{2}$ and ${T}_{2}^{*}$, leads to linear equations

3 $$S_{GE} \left( {t_{GE} } \right) = A_{GE} \left( {t_{GE} ,T_{2}^{*} } \right) \cdot \Omega_{GE} \left( {T_{2}^{*} } \right)$$

4 $$S_{SE} \left( {t_{SE} } \right) = A_{SE} \left( {t_{SE} ,T_{2} } \right) \cdot \Omega_{SE} \left( {T_{2} } \right)$$

where ${A}_{GE}$ and ${A}_{SE}$ are operator matrices solving the respective forward problems, ${S}_{GE}$ and ${S}_{SE}$ are signal vectors at respective echo times ${t}_{GE}$ and ${t}_{SE}$, and ${\Omega }_{GE}$ and ${\Omega }_{SE}$ are the relaxation spectra vectors on a suitable grid of relaxation times, ${T}_{2}$ and ${T}_{2}^{*}$, respectively. The forward operators, ${A}_{GE}$ and ${A}_{SE}$, can be precomputed for efficiently solving the nonlinear inverse problem.

### Myelin-induced frequency shift: extension to a complex ME-GE signal model

The ME-GE exponential signal model of Eq. (1) does not account for compartmental frequency shifts within the imaging voxel. It was previously shown that susceptibility-induced frequency shifts give rise to a non-exponential ME-GE signal [16], resulting in biased MWF estimates from ME-GE magnitude data. Therefore, we extend the ME-GE signal model to account for frequency shifts, $\Delta \omega$, in the MW and AEW compartments. Following the approach from Nam et al. [7], we extend Eq. (3) to a complex signal model by introducing a complex forward operator

5 $${A}_{GE}\left(t,{T}_{2}^{*},\Delta \omega ,\Delta {\Phi }_{0}\right)=\text{exp}\left[-t/{T}_{2}^{*}\right]\text{exp}\left[-{\text{i}}\left(\Delta \omega t+\Delta {\Phi }_{0}\right)\right]\hspace{0.17em}$$

where $\Delta {\Phi }_{0}$ is a spatially dependent constant phase offset. In the following parameterized forward model, we assume constant frequency shifts, $\Delta {\omega }_{1}$ and $\Delta {\omega }_{2}$, for the MW and AEW compartments, respectively [7, 16].

### Inversion of bi-Gaussian parametric models

We first present the method for the single inversion, i.e., the method to recover the MWF from ${T}_{2}$ (respectively ${T}_{2}^{*}$) data only, before describing the joint inversion. To model the ${T}_{2}$ (respectively ${T}_{2}^{*}$) distribution, we resort to a parametric model, assuming the presence of two compartments with different ${T}_{2}$ values, one for the MW pool (fast compartment) and one for axonal/extra-cellular water pool (slow compartment). Similar to [6], we propose a bi-Gaussian parametric model, which strongly improves the optimization stability compared to a bi-impulse (delta function) model.

Within this assumption, the ${T}_{2}$ distribution is modeled by six parameters: the ${T}_{2}$ means (${\mu }_{1}$, ${\mu }_{2}$) and standard deviations (${\sigma }_{1}$, ${\sigma }_{2}$) of both compartments, the integral of the axonal/extra-cellular compartment (${I}_{2}$), and finally the $MWF$, which is the ratio of the integral of the myelin compartment over the sum of the two compartment integrals, ${I}_{1}/\left({I}_{1}+{I}_{2}\right)$. Most references in the literature, using either ${T}_{2}$ or ${T}_{2}^{*}$ parametric models, first invert for all compartment integrals (or amplitudes if delta functions are used instead of Gaussian functions) and based on that compute MWF [2, 7, 17]. Instead, we used the MWF directly as one of the model parameters for the inversion. We found that this parametrization leads to a slightly better resolution and spatial continuity on the final 2D MWF map than inverting for both compartment integrals separately.

Let $G\left(\tau |\mu ,\sigma ,I\right)$ be a Gaussian function over the variable $\tau$, defined by its mean value $\mu$, standard deviation $\sigma$ and integral value $I$. Denoting $x=\left({\mu }_{1},{\sigma }_{1},MWF,{\mu }_{2},{\sigma }_{2},{I}_{2}\right)$ as the model vector i.e., the six parameters describing the two Gaussian functions, the ${T}_{2}$ distribution is given by

6 $${\Omega }_{SE}\left({T}_{2}|x\right)=G\left({T}_{2}|{\mu }_{1},{\sigma }_{1},{I}_{1}\right)+G\left({T}_{2}|{\mu }_{2},{\sigma }_{2},{I}_{2}\right)\hspace{0.33em}$$

where ${I}_{1}$ and ${I}_{2}$ are the integrals of the MW pool and the axonal/extra-cellular water pool (AEW), respectively. ${I}_{1}$ is not treated as a model parameter, but expressed according to the MWF definition as ${I}_{1}={I}_{2}MWF/\left(1-MWF\right)$. Let ${S}_{SE}\left(t\right)$ be the observed ME-SE signal with $t$ being the echo times vector. For a single inversion, the unregularized minimization problem is then given by

$$\mathop {\min }\limits_{x} \;\left\| {S_{SE} \left( t \right) - \hat{S}_{SE} \left( {t|x} \right)^{2} } \right\|$$

where ${\widehat{S}}_{SE}\left(t|x\right)={A}_{SE}\left(t,{T}_{2}\right)\cdot {\Omega }_{SE}\left({T}_{2}|x\right)$ is the parameterized forward model from the ${T}_{2}$ relaxometry distribution to the data space (ME-SE decay curve).

Since the problem is highly ill-posed, regularization has to be added to stabilize the inversion. However, classical Tikhonov regularization aims to minimize the model parameters and, therefore, results in low and unrealistic values for the myelin and axonal/extra-cellular ${T}_{2}$ values (${\mu }_{1}$ and ${\mu }_{2}$). To overcome this problem, we added a constraint on the model to stay as close as possible to the initial model, but weighting this constraint mainly on the mean of the parameters. The regularized minimization problem is given by

7 $$\mathop {\min }\limits_{x} \left\| \;S_{SE} \left( t \right) - \hat{S}_{SE} \left( {t|x} \right)\right\|^{2} + \left\| \lambda \cdot \left( {x - x_{in} } \right)\right\|^{2}$$

where ${x}_{\text{in}}$ is the initial model vector (cf. Table 1) and $\lambda$ a model-sized regularization vector. We resort to this regularization for the fast and slow compartments mean values (${\mu }_{1}$,${\mu }_{2}$) since we do have prior information on these parameters from the literature, and want to use this knowledge as initial values to better constraint the inversion. Regarding the standard deviations, which are initialized with small values (see Tab. 1), this regularization helps to maintain the values as small as possible, since we would expect delta functions in an ideal noise-free world. Finally, since we do not have knowledge on the MWF and slow compartment integral ($MWF$ and ${I}_{2}$), we set the corresponding elements of $\lambda$ for these two parameters to negligibly small, non-zero values (see Tab. 1).

**Table 1:** Summary of the model parameters with their initial values (i.v.) and upper and lower bound, respectively

#	Parameter	Description	Unit	i.v	Bounds	Regularization
1	${\mu }_{1,{T}_{2}}$	${T}_{2}$ mean of the MW distribution	[ms]	18	[5, 35]	${\lambda }_{1/1}^{S/J}$=0.02
2	${\sigma }_{1,{T}_{2}}$	${T}_{2}$ standard deviation of the MW distribution	[ms]	0.1	[0.1, 5]	${\lambda }_{2/2}^{S/J}$=0.01
3	${\mu }_{2,{T}_{2}}$	${T}_{2}$ mean of the AEW distribution	[ms]	80	[45, 180]	${\lambda }_{3/3}^{S/J}$=0.02
4	${\sigma }_{2,{T}_{2}}$	${T}_{2}$ standard deviation of the AEW distribution	[ms]	0.1	[0.1, 5]	${\lambda }_{4/4}^{S/J}$=0.01
5	${I}_{2,{T}_{2}}$	Integral of the ${T}_{2}$ AEW distribution	–(normalized)	2	[0.1, 5]	${\lambda }_{5/5}^{S/J}$=0.01
6	$MWF$	Myelin-water fraction	–(normalized)	0.1	[0, 0.85]	${\lambda }_{6/6/6}^{S/G/J}$=0.01
7	${\mu }_{1,{T}_{2}^{*}}$	${T}_{2}^{*}$ mean of the MW distribution	[ms]	10	[5, 25]	${\lambda }_{1/7}^{G/J}$=0.02
8	${\sigma }_{1,{T}_{2}^{*}}$	${T}_{2}^{*}$ standard deviation of the MW distribution	[ms]	0.1	[0.1, 5]	${\lambda }_{2/8}^{G/J}$=0.01
9	$\Delta {\omega }_{1}$	Frequency shift of the MW component	[Hz]	−5	[−75, 75]	${\lambda }_{3/9}^{G/J}$=0.02
10	${\mu }_{2,{T}_{2}^{*}}$	${T}_{2}^{*}$ mean of the AEW distribution	[ms]	60	[55, 180]	${\lambda }_{4/10}^{G/J}$=0.02
11	${\sigma }_{2,{T}_{2}^{*}}$	${T}_{2}^{*}$ standard deviation of the AEW distribution	[ms]	0.1	[0.1, 5]	${\lambda }_{5/11}^{G/J}$=0.01
12	${I}_{2,{T}_{2}^{*}}$	Integral of the ${T}_{2}^{*}$ AEW distribution	– (normalized)	1	[0.1, 5]	${\lambda }_{7/12}^{G/J}$=0.01
13	$\Delta {\omega }_{2}$	Frequency shift of the AEW component	[Hz]	0	[−75, 75]	${\lambda }_{8/13}^{G/J}$=0.01
14	${\Phi }_{0}$	Constant phase offset	[rad]	0	[0, 2 $\pi$]	${\lambda }_{9/14}^{G/J}$=0.01

The rows 1–6 (until $\text{MWF}$) denote parameters for single ME-SE inversion, and the rows 7–14 for single ME-GE inversion, while joint inversion uses all parameters. The last column provides the $\uplambda$ regularization vectors used in the simulations. The superscripts ${\uplambda }^{\text{S}}$, ${\uplambda }^{\text{G}}$, and ${\uplambda }^{\text{J}}$ denote vectors for single ME-SE inversion, single ME-GE inversion, and joint inversion, respectively. The subscripts give the number of the element in the corresponding regularization vector, and the values correspond the regularization used for the in vivo data, while the simulations used $\uplambda =0.01$ for all parameters

Accordingly, the above equations also apply for the inversion of magnitude ME-GE data, where the model vector $x$ contains the respective six parameters of the ${T}_{2}^{*}$ distribution. Incorporating the complex signal model from Eq. (5), the previous equation naturally extend for the inversion of complex ME-GE data:

8 $$\mathop {\min }\limits_{x} \left\|S_{GE} \left( t \right) - \hat{S}_{GE} \left( {t|x} \right)\right\|^{2} + \left\|\lambda \cdot \left( {x - x_{in} } \right)\right\|^{2}$$

with the parameterized complex signal forward model

9 $${\widehat{S}}_{GE}\left(t|x\right)=\left[G\left({T}_{2}^{*}|{\mu }_{1},{\sigma }_{1},\frac{{I}_{2}\hspace{0.17em}MWF}{1-MWF}\right){\text{e}}^{-t/{T}_{2}^{*}+{\text{i}}\Delta {\omega }_{1}t}+G\left({T}_{2}^{*}|{\mu }_{2},{\sigma }_{2},{I}_{2}\right){\text{e}}^{-t/{T}_{2}^{*}+{\text{i}}\Delta {\omega }_{2}t}\right]{\text{e}}^{{\text{i}}{\Phi }_{0}}$$

where the model vector $x=\left({\mu }_{1},{\sigma }_{1},\Delta {\omega }_{1},MWF,{\mu }_{2},{\sigma }_{2},{I}_{2},\Delta {\omega }_{2},\Delta {\Phi }_{0}\right)$ now contains nine values for the parameterization, including the MW and AEW frequency shifts, $\Delta {\omega }_{1}$ and $\Delta {\omega }_{2}$, and the constant phase term $\Delta {\Phi }_{0}$.

In contrast to the single inversions, the joint inversion aims to find simultaneously distributions for both ${T}_{2}$ and ${T}_{2}^{*}$ which fit the respective decay curves, with the same $MWF$ value in both distributions. The model vector $x$ has now 14 parameters since it represents two signals, the SE signal modeled by six parameters and the GE signal modeled by nine parameters, but the $MWF$ parameter is the same in both. Then, the minimization problem of the joint inversion can be stated as the sum of both residuals with a regularization term (analog to the single inversions) using 14 parameters:

10 $$\mathop {\min }\limits_{x} \,\,\,\alpha\left\| S_{SE} \left( {t_{SE} } \right) - \hat{S}_{SE} \left( {t|x} \right)\right\|^{2} + \left\|S_{GE} \left( {t_{GE} } \right) - \hat{S}_{GE} \left( {t|x} \right)\right\|^{2} + \left\|\lambda \cdot \left( {x - x_{in} } \right)\right\|^{2}$$

where ${t}_{GE}$ denote the echo times of the ME-SE and ME-GE acquisitions, respectively, and $\alpha$ is a scalar weighting factor to adjust the influence of the first term, the ME-SE data consistency. Since ME-GE acquisition has the double amount of data (magnitude and phase) than the ME-SE acquisitions (magnitude only), $\alpha =2$ was chosen for equal weighting of both terms for all simulations and in vivo experiments.

## Methods

### Single and joint inversion implementation

We iteratively solve the nonlinear inversion problems from Eqs. (7–10) for single and joint inversion with a local-search optimization method, the Levenberg–Marquardt algorithm [18]. At each iteration, we compute the Fréchet derivatives by finite difference. We solve the linearized problem as a classical linear least-squares problem with bounds on the variables, using the *lsq_linear* method from the Scipy Python library. We added a damping term (value 0.01) to the minimization for more stability. Table 1 summarizes the parameters to be inverted (cf. model vector $x$ of Eq. (10)), their initial values, and the upper and lower bounds during minimization. Note that the joint inversion does not explicitly enforce ${T}_{2}^{*}\le {T}_{2}$ during the optimization but implicitly via the forward models in combination with realistic data. With the inversion parameters in Table 1, such a physically impossible solution was never observed for all simulated and in vivo data.

### Simulations

Synthetic ME-GE and ME-SE data were generated to test the inversion approaches in simulations with known ground truth. We used a published MW fraction atlas [19] to compute voxel-wise two-pool Gaussian relaxation spectra. The Gaussian integrals were modified to match the respective $MWF$ value per voxel. The simulations were repeated six times with different parameters for the Gaussian distributions, which were randomly drawn within certain ranges: ${\mu }_{1,{T}_{2}}\in \left[16,22\right]$ ms, ${\mu }_{2,{T}_{2}}\in \left[65,80\right]$ ms, ${\mu }_{2,{T}_{2}^{*}}\in \left[10,15\right]$ ms, ${\mu }_{2,{T}_{2}^{*}}\in \left[48,62\right]$ ms, and ${\sigma }_{1/2,{T}_{2}}/{\sigma }_{1/2,{T}_{2}}\in \left[0.2,1\right]$ ms (cf. Table 1). The compartmental frequency shifts for ME-GE signal simulations were modeled accordingly with $\Delta {\omega }_{1}\in \left[-2,-10\right]$ Hz, $\Delta {\omega }_{2}\in \left[-3,3\right]$ Hz, and $\Delta {\Phi }_{0}\in \left[0,\pi \right]$. We chose these ranges according to the literature and to ensure ${T}_{2}^{*}\le {T}_{2}$. All other distribution parameters were chosen constant but not too close to the initial model.

**Fig. 1:**
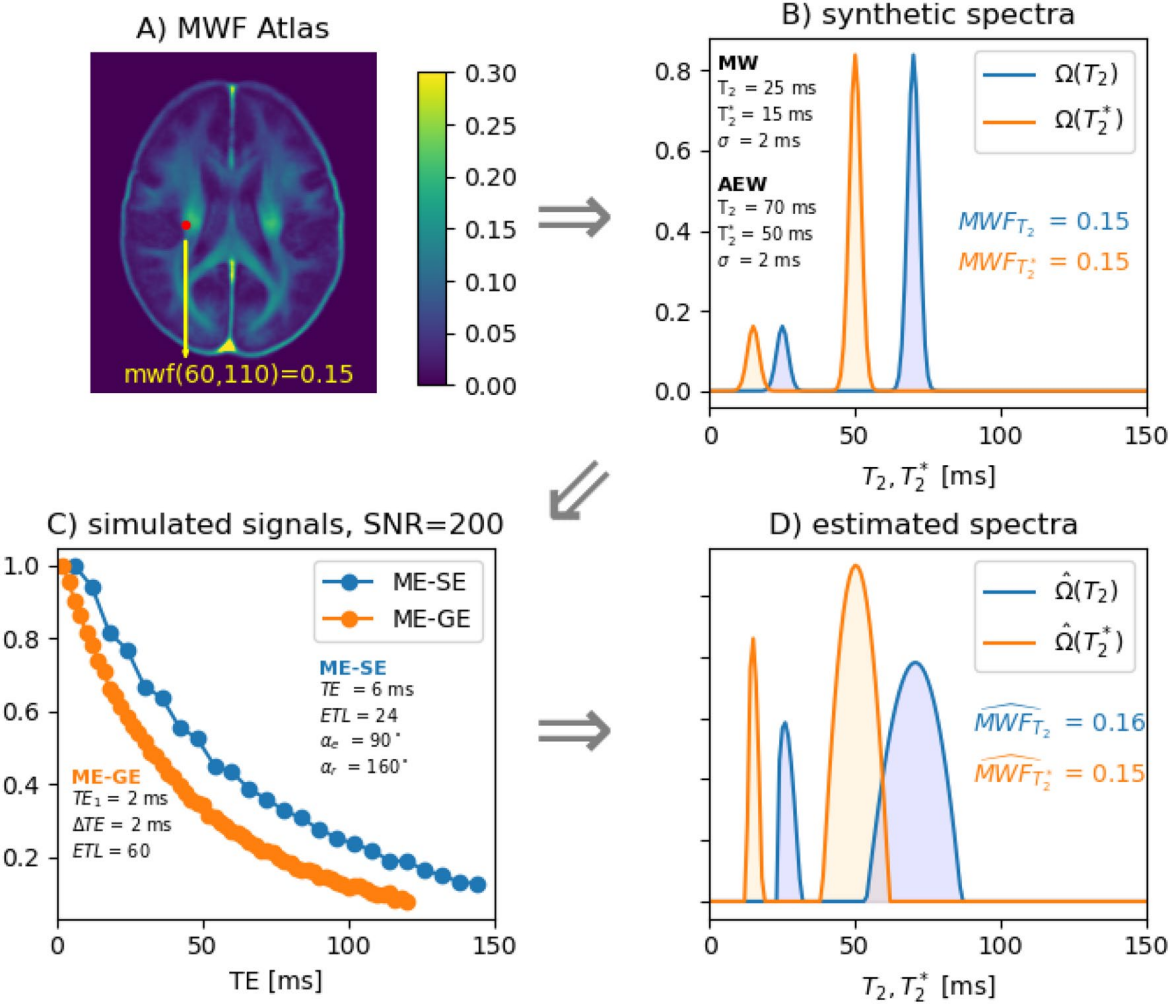
Workflow of the simulation process. For each voxel from one slice of the MWF atlas (**A**) synthetic relaxation spectra are generated (**B**) with realistic values for the respective Gaussians. Next, ME-GE and ME-SE signals are simulated by applying forward models and adding noise (**C**). Finally, the parametric fitting of the individual inversions provides the estimated spectra and resulting MWF estimates (**D**)

From the spectra we computed the ME-GE and ME-SE signals with the respective forward models. The sequence parameters are given in Fig. 1 C: first echo time ($T{E}_{1}$), increment ($\Delta TE$), echo train length (ETL), and ME-SE flip angles (${\alpha }_{e}$ and ${\alpha }_{r}$). After adding Gaussian noise to the real and imaginary part of all signals (SNR = 150 for the first $TE$) to the signals, individual inversions and joint inversion based on the Levenberg–Marquardt algorithm were compared. For the simulation the same regularization term (0.01) was used for all $\lambda$-parameters (cf. Table 1). The workflow for the simulation process is schematically depicted in Fig. 1.

### Data acquisition

Measurements were conducted at a 3T MAGNETOM Skyra scanner (Siemens Healthineers) using custom slab selective 3D sequences with 2-mm isotropic resolution to ensure sufficiently high SNR and short echo times. Both sequences, ME-GE and ME-SE, encode the identical FOV of 220 × 220x48 mm^3^. While the in-plane axial FOV covers complete transverse slices, the FOV in head-feet direction (48mm) was chosen to cover several important WM structures for region of interest (ROI) analysis (cf. Result section). Furthermore, the repetition times of the ME-GE and ME-SE sequences were matched to $TR$=900 ms. This is an essential requirement for the analysis, which does not take ${T}_{1}$ relaxation into account.

For time-efficient ME-GE acquisition, we used a custom multi-echo 3D-EPI sequence [20] as a monopolar ME-GE sequence with ramp sampling by setting the EPI factor to 1 and disabling phase correction and echo time shifting. We allowed the sequence to acquire non-equidistant echo times. In order to best capture the MW signal component at short echo times and at the same time minimize the gradient duty cycle, 32 echoes with exponentially increasing spacing were acquired:$\Delta T{E}_{n}=\Delta T{E}_{0}{\text{e}}^{nr\hspace{0.17em}\Delta T{E}_{0}}\hspace{0.33em},n=1,...,31$. Consequently, echo times increased according to $T{E}_{n}=T{E}_{0}+\Delta T{E}_{0}\left({\text{e}}^{nr\hspace{0.17em}\Delta T{E}_{0}}-1\right)/\left({\text{e}}^{r\hspace{0.17em}\Delta T{E}_{0}}-1\right)$. Using $T{E}_{0}$=2 ms, $\Delta T{E}_{0}$=1.5 ms and increase rate $r$=0.02, the resulting echo times were: $T{E}_{GE}$=[2, 3.5,…, 73.89,77.58] ms. Further sequence parameters were: $TR$=900 ms, 60° nominal flip angle, 1516 Hz/pixel readout bandwidth, CAIPIRINHA $2\times {2}_{z1}$ parallel imaging, $TA$=9:08 min (total acquisition time).

**Fig. 2:**
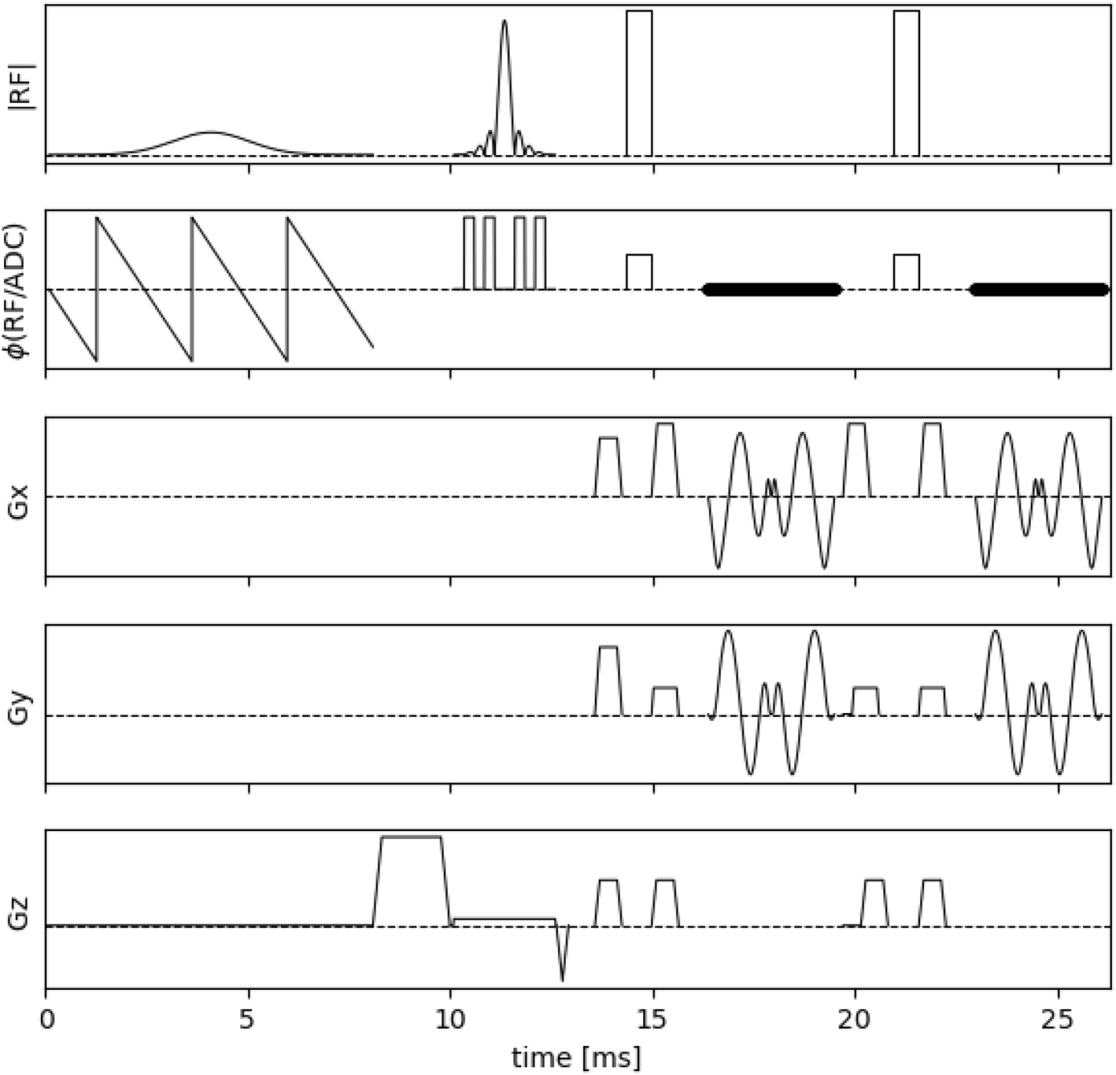
Diagram of the Pulseq implementation for the ME-SE 3D imaging sequence with in-plane segmented spiral-in/out readout (Gx,Gy) and through-plane phase encoding (Gz). The plot depicts the fat suppression, slab selective excitation, and the first two echoes of the CPMG train with nonselective refocusing pulses. After 24 echoes a fill-time is appended to result in $\text{TR}$=900 ms. This sequence is repeated in a double loop structure acquiring all spiral interleaves (inner loop) and phase encoding steps (outer loop)

The total acquisition time of a purely phase-encoded 3D ME-SE sequence would be too long for in vivo applications [21]. Therefore, we implemented a ME-SE sequence with a segmented spiral-in/out readout using the Pulseq [22] framework. The sequence diagram is depicted in Fig. 2: after fat suppression and slab selective excitation, a CPMG echo train is applied with nonselective refocusing pulses in order to avoid slice profile effects (which would confound the EPG-based MWF analysis). The spiral readout of the CPMG echo train is strongly segmented to ensure short echo times. Image reconstruction was performed with the BART toolbox [23] using an established open‐source MR imaging and reconstruction workflow [24], including correction of spiral trajectory imperfections by means of the GIRF approach [25]. For the in vivo acquisitions the following sequence parameters were chosen, $T{E}_{SE}$=6.6 ms (CPMG echo spacing), $ETL$=24, $TR$=900 ms (repetition time), 50° nominal excitation flip angle, $TA$=7:48 min (total acquisition time). These parameters could be achieved utilizing ${N}_{s}$=21 spiral interleaves and ${N}_{z}$=24 phase encoding steps along the slab dimension.

The slightly lower flip angle of ME-SE (50°) compared to ME-GE (60°) ensures the same ${T}_{1}$-saturation for $TR$=900 ms, assuming ${T}_{1}\approx$ 900 ms for the AEW component. It is difficult to measure ${T}_{1}$ of MW, but it is assumed to be much shorter than the ${T}_{1}$ of the AEW component [3, 26]. For instance, ${T}_{1}=118$ ms was reported for MW at 3T [27]. Thus, we assume full recovery of the MW component during $TR$=900 ms for both sequences. In total, this ensures equal (apparent) $MWF$ in ME-GE and ME-SE acquisitions, which is a central requirement for the joint inversion approach.

Both sequences were acquired twice in order to use two-fold averaged data for the single ${T}_{2}$ and ${T}_{2}^{*}$ inversions, whereas the joint inversion is based on single ME-GE and ME-SE acquisitions. This way approximately the same amount of data and scan time enters all three inversion approaches.

As a first scan in the experiment, a flip angle (B1) map is acquired with the 3DREAM sequence [28] as required for the EPG signal modeling. The 3DREAM acquisition parameters were: FOV = 220 × 220x160 mm^3^, isotropic resolution of 5 mm, $TA$ = 1:41 min. Finally, a custom MP-RAGE sequence [29] with 2D acceleration capabilities [30] was acquired for image registration and segmentation, enabling ROI analysis of the $MWF$ estimates. The MP-RAGE acquisition parameters were: FOV = 256 × 256x172 mm^3^, isotropic resolution of 1 mm, $TA$ = 3:12 min. The total acquisition time of the entire in vivo protocol was approximately 40 min. Three healthy subjects (age range 34 $\pm$ 11 years) were scanned after providing informed consent in accordance with local institutional review board regulations.

### Data analysis

A custom data analysis pipeline was implemented in Python. First, the B1 map was registered to the ME-GE/ME-SE images with the *AntsPy* library. Voxel-wise single and joint inversions were performed as described above. For the single inversions, the average of the two respective ME-GE and ME-SE acquisitions was used, whereas the joint inversion was applied to single acquisitions (the first one for both, ME-GE and ME-SE). The subjects were highly compliant and we did not observe any misalignment between the datasets. Therefore, in order to avoid re-slicing errors, image registration was neither performed between ME-GE and ME-SE data (for joint inversion) nor between repeated scans (for single inversions). However, averaging of the complex ME-GE data required phase-matching between the two scans to account for local frequency and phase drifts, e.g., due to gradient heating. To this end, a voxel-wise linear fit of the TE-dependent phase change between repeated ME-GE scans was performed and then subtracted from the phase of the repeated scan before complex-valued averaging.

Single and joint inversions were performed voxel-by-voxel as described above. The inversion of ME-GE data and the joint inversion were run twice, once with the suggested complex signal model (including compartmental frequency shifts), and once with the simplified mono-exponential decay model. Here, the ME-GE inversion problem is similar to Eq. (7) for ME-SE data, i.e., only the six parameters of the ${T}_{2}^{*}$ are estimated. (Likewise, the joint inversion reduces to 9 parameters instead of 14, cf. Table 1.) To ensure equal weighting for the joint inversion, ME-GE and ME-SE data were normalized prior to the analysis, ${S}_{GE/SE}\left(T{E}_{min}\right)\equiv 1$. A slightly higher regularization for the parameters of the MW component was chosen in comparison to the simulation (cf. Table 1). However, again the same regularization parameters were applied in single and joint inversions. This slight increase in regularization showed more homogeneous MWF results for all three inversion methods (comparison not shown), potentially counteracting a model-induced bias for real data.

Probability GM and WM maps were generated from the MP-RAGE with SPM12 (MATLAB vR2022a) [31]. Only voxels with a probability higher than 95% were included to create the respective masks. Registration from the MP-RAGE to the ME-SE and ME-GE spaces were performed with *ANTs* [32] using a rigid transformation, which was then applied to the GM and WM masks. To obtain WM tract ROIs, we used the JHU ICBM-DTI-81 WM atlas [33]. The MNI 152 T1 template was registered to the ME-GE and ME-SE spaces with *ANTs* using a symmetric normalization (SyN), which was then applied to the atlas. Ten regions were included in the analysis: total WM, ALR (anterior limb of the internal capsule—right), ALL (anterior limb of the internal capsule—left), PLR (posterior limb of the internal capsule—right), PLL (posterior limb of the internal capsule—left), GCC (genu of the corpus callosum), BCC (body of the corpus callosum), SCC (splenium of the corpus callosum), AF (anterior forcepts) and PF (posterior forcepts). Parameter estimation was performed in the native space. Finally group statistics across all ROI voxels and subjects were computed to obtain mean and standard deviation of the estimated model parameters.

## Results

### Simulation results

**Fig. 3:**
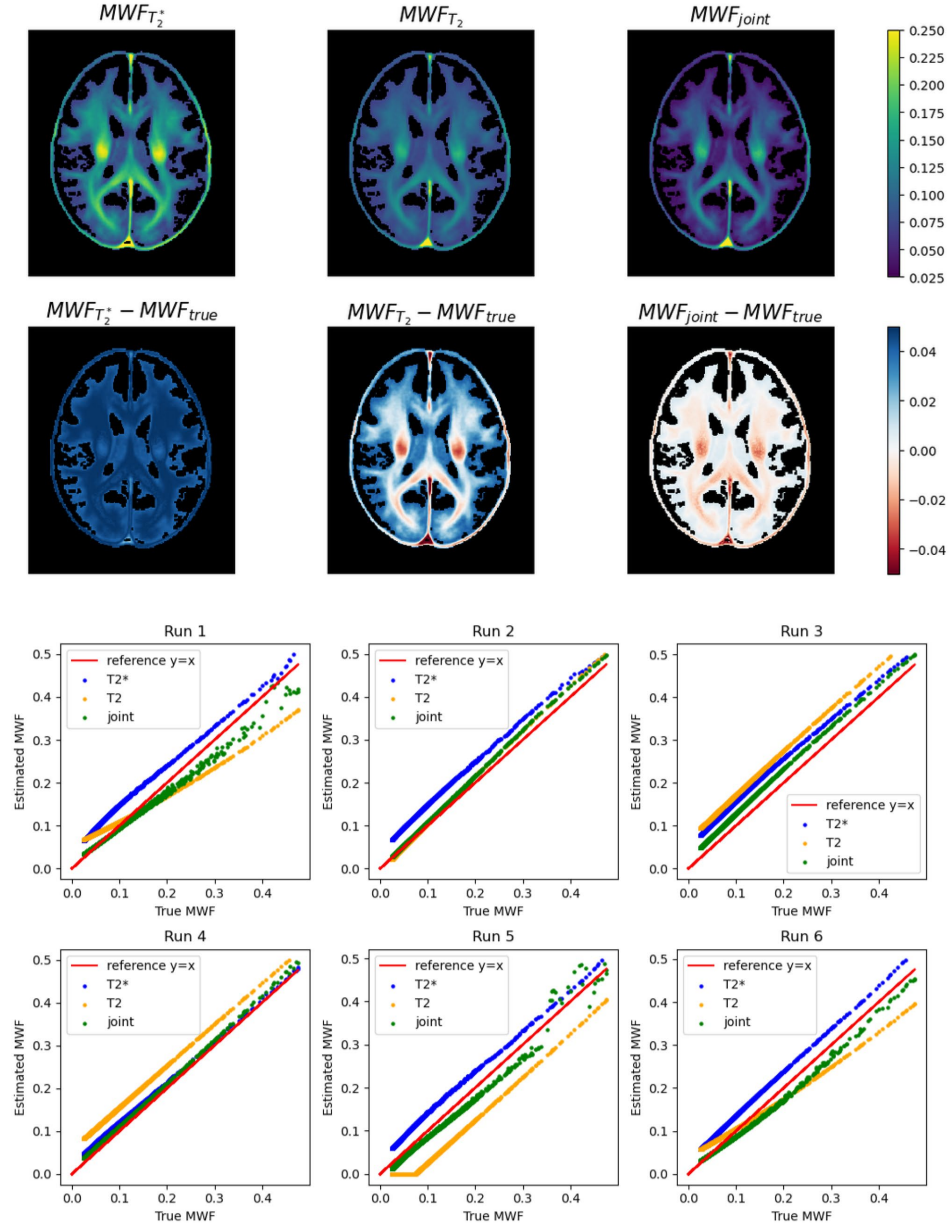
Depiction of the simulation results. The top row of images shows the estimated MWF maps from one simulation run of the single (${\text{T}}_{2}$ and ${\text{T}}_{2}^{*}$) inversions and the joint inversion. The second row of images show the respective deviations to the ground truth. The last two rows show scatter plot for all simulation runs displaying the deviation between the estimated MWF and the true MWF. The data points show the MWF values in the individual voxels for the repeated simulation runs and within the (discontinuous) range of MWF values present in the depicted slice of the atlas

The top row in Fig. 3 depicts $MWF$ maps resulting from from one simulation run of the single inversions (${T}_{2}$ and ${T}_{2}^{*}$) and the joint inversion. To emphasize the comparison with the $MWF$ atlas (ground truth), the second row shows the difference maps, where the sign indicates if the $MWF$ was over- or underestimated. It can be seen that the joint inversion $MWF$ estimate is closer to the ground truth than the single inversions. The last two rows of Fig. 3 depict cross-plots for all six simulation runs, displaying the voxel-wise estimated $MWF$ values against the ground truth. The closer the points to the identity ($x=y$), the more accurate the inversion. For example, in Run 5 the ${T}_{2}^{*}$ inversion overestimates the $MWF$ and the ${T}_{2}$ inversion underestimates the $MWF$, while the joint inversion lies in between. In Run 3 all inversions overestimate the $MWF$, but the joint inversion is closest to the ground truth. When a single inversion provides a good estimate, then the joint inversion is close to these values (Runs 2 and 4). In general, the plots show that the joint inversion is closer to the ground truth than the single inversions for almost all data points. In most cases, the observed error of the $MWF$ estimates resulted from deviations in the estimated integrals (${I}_{1},{I}_{2}$), while the estimates of Gaussian mean and standard deviation was always very close to the ground truth.

### In-vivo results

**Fig. 4:**
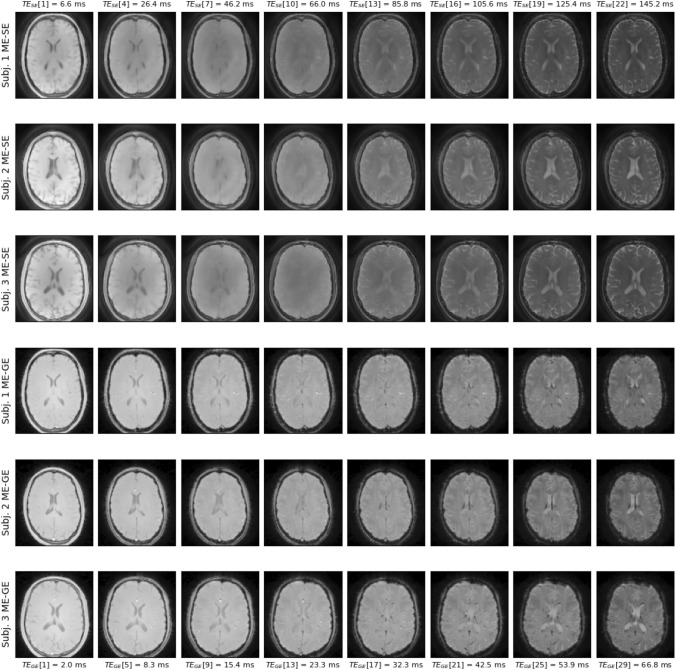
Single slice imaging data from all three subjects. The first three rows show ME-SE images and the last three rows show ME-GE images. The columns show the signal decay at eight echo times (TEs) over the entire range of the echo train. The TEs of ME-SE and ME-GE are given in the labels at the top and at the bottom, respectively. Note that ${\text{TE}}_{\text{GE}}^{\text{max}}\ll {\text{TE}}_{\text{SE}}^{\text{max}}$

**Fig. 5:**
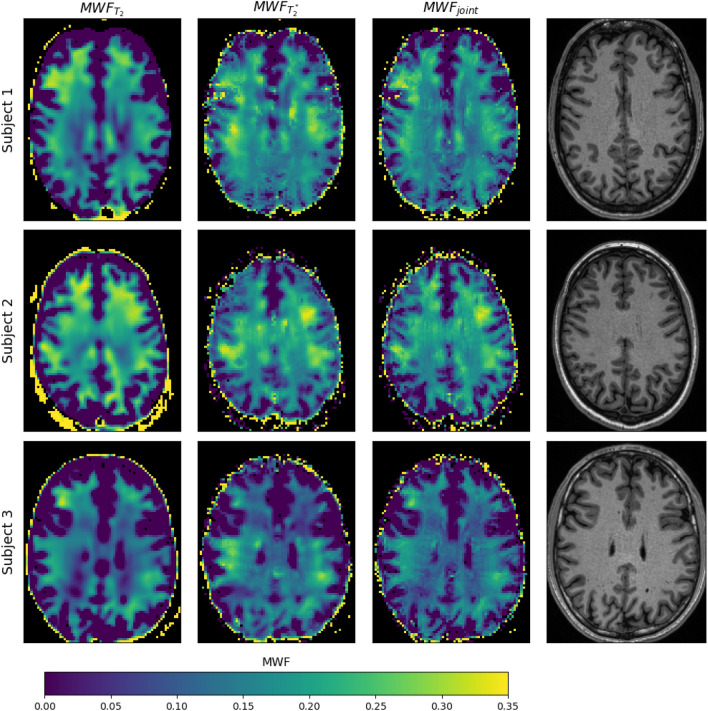
MWF results for all subjects for single inversions (first and second column) and for the joint inversion (third column). The last column shows the respective slice of the ${\text{T}}_{1}$-weighted scan for anatomical reference. For all three inversion methods the estimated MWF values are consistently higher in WM than in GM and CSF and lie in the expected range known from the literature. The joint inversion maps appear less blurred and show less variation within in WM as compared to the maps from the single inversions

**Fig. 6:**
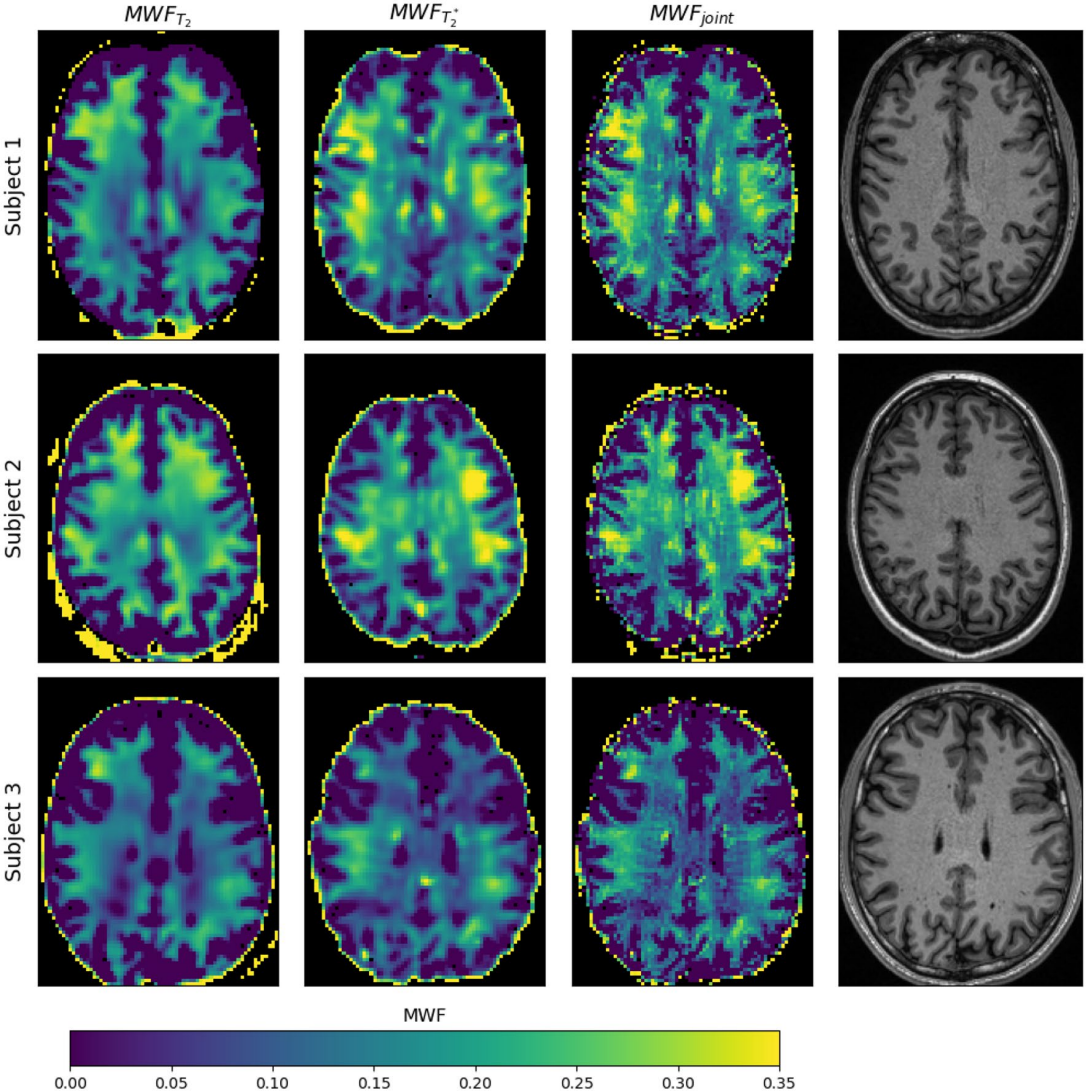
Same display of MWF results as in Fig. 5 but without taking compartmental frequency shifts into account for the ${\text{MWF}}_{{\text{T}}_{2}^{*}}$ and the ${\text{MWF}}_{\text{joint}}$ fit. (The ${\text{MWF}}_{{\text{T}}_{2}}$ fit is exactly the same as in Fig. 5 and again displayed for comparison.) Regionally high MWF values can be observed in the ${\text{MWF}}_{{\text{T}}_{2}^{*}}$-map, which also transfers to the ${\text{MWF}}_{\text{joint}}$ map. This is especially pronounced for subjects 1 and 2 and might be attributed to frequency shifts in white matter fiber bundles

**Fig. 7:**
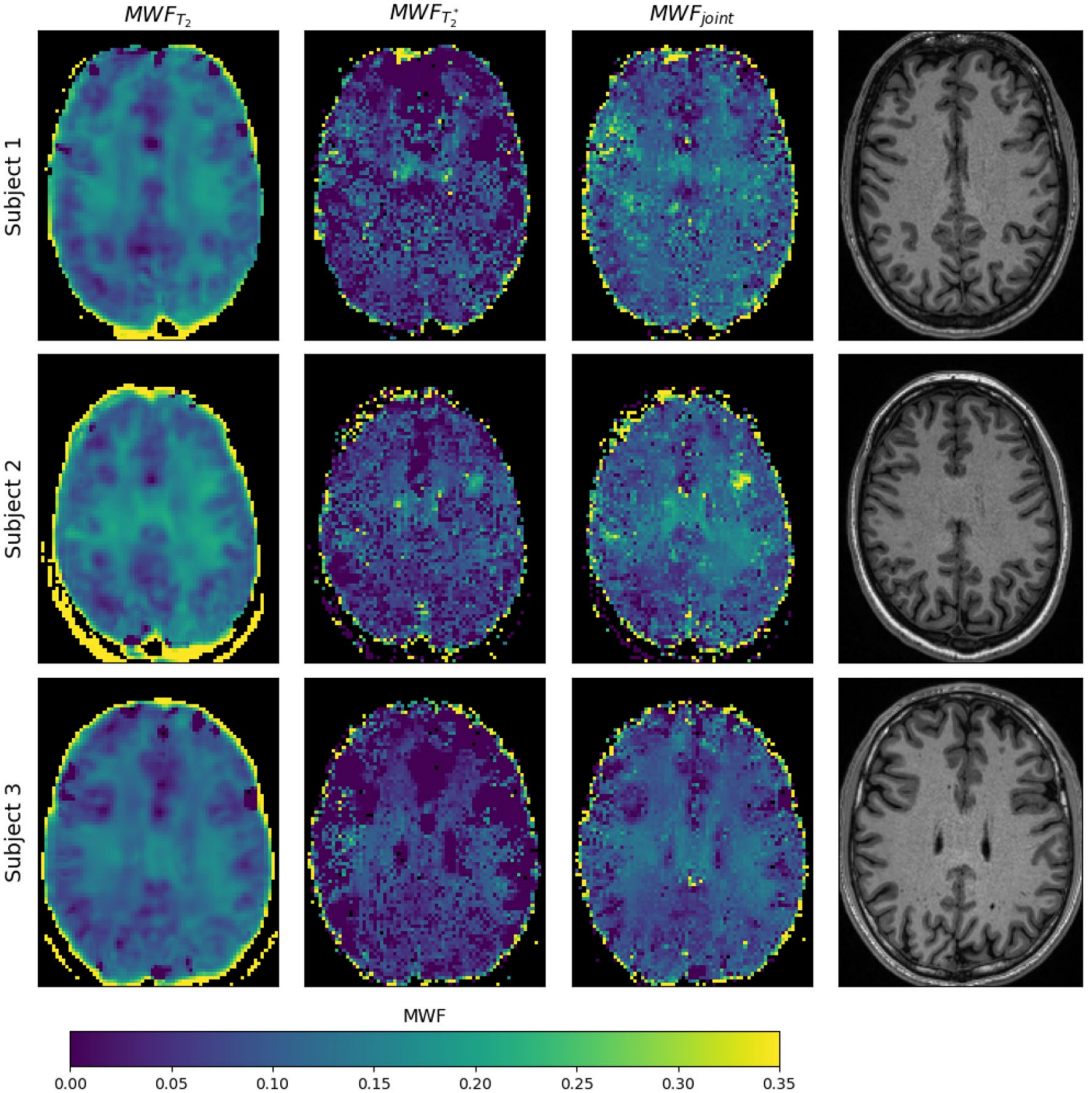
Same display of MWF results as in Fig. 5 but using low regularization in the inversion ($\lambda =0.005$ for all model parameters). While ${\text{T}}_{2}$ inversion is less affected, the ${\text{T}}_{2}^{*}$ result is dominated by noise, which also takes over to the joint inversion results

The in vivo results shown in this section depict a single representative slice for each of the three subjects. Fly-through movies showing the quantitative maps for all slices (and subjects) are provided as Supporting Information Videos S1-2. Figure 4 shows the acquired ME-GE and ME-SE imaging data for all subjects and a subset of all echo times (see figure legend). The images are free of obvious artifacts and generally good image quality was observed in all cases. The image SNR of the first echo was in the range of 200 for both sequences and all subjects. The $MWF$ results of the single inversions (${T}_{2}$ and ${T}_{2}^{*}$) and the joint inversion are shown in Fig. 5 for all subjects. All inversion methods show increased $MWF$ values in WM compared to GM, as expected. The most obvious differences between single and joint inversion results are the reduced blurring and overall more distinct contours in the joint inversion $MWF$ maps, which results in sharper depiction of WM structures. This effect becomes even more evident when only the ME-GE magnitude data is used for the inversion, as shown in Fig. 6. Here, the $MWF$-map from the single ${T}_{2}^{*}$ inversion is more blurred as compared to corresponding map in Fig. 5. Again, the joint inversion produces the most detailed $MWF$ map. However, large $MWF$ values potentially arising from frequency-shift induced signal changes in WM are present in both maps. Finally, the effect of regularization is shown in Fig. 7. Here, the results were obtained with low regularization for all model parameters ($\lambda$=0.005), leading to very low-quality $MWF$ maps for the single ${T}_{2}^{*}$ inversion. This underlines the need for regularization, also in the case of joint inversion.

A quantitative evaluation of all estimated parameters is presented in Table 2, showing group averaged fit results in several WM ROIs, which are present in the acquired 3D slab. Interestingly, the joint inversion method often produces $MWF$ mean values that are higher than those observed from inverting the ME-SE and ME-GE datasets separately. The group standard deviations, given in brackets in Table 2, indicate that there are only small differences between subjects for all estimated parameters, especially for the $MWF$. This quantitative observation, which holds for single and joint inversions, may not be visually supported when inspecting Fig. 5. Here, the colorbar scaling was chosen to enhance regional differences in $MWF$, which also visually enhances variation between subjects. Finally, the correlations between the estimated parameters are provided as supporting information S3 for single and joint inversions, showing that parameter estimates are mostly independent of each other (Fig. 7).

**Table 2:**
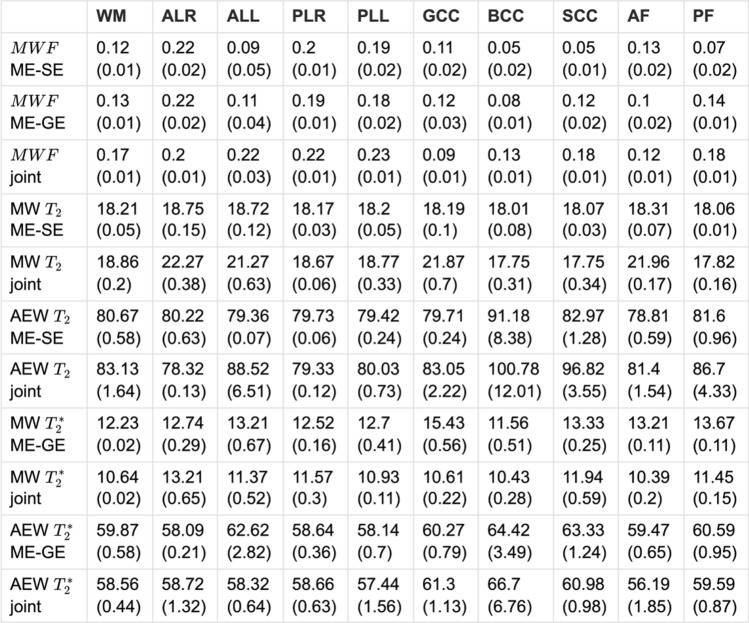
Group statistics of the quantitative fit results (rows) for several ROIs (columns): *WM* (white matter), *ALR* (anterior limb of the internal capsule—right), *ALL* (anterior limb of the internal capsule—feft), *PLR* (posterior limb of the internal capsule—right), *PLL* (posterior limb of the internal capsule—left), *GCC* (genu of the corpus callosum), *BCC* (body of the corpus callosum), *SCC* (splenium of the corpus callosum), *AF* (anterior forcepts), *PF* (posterior forcepts)

Each row shows the group mean value in the ROI and below the standard deviation in brackets. The first three rows compare the MWF estimates for single and joint inversions. The next eight rows show ${\text{T}}_{2}$ and ${\text{T}}_{2}^{*}$ estimates of the MW and the AEW component for the respective inversion approaches

## Discussion

MWI is a practical approach for indirect in vivo quantitative mapping of brain myelin content, an important marker for understanding brain development, aging, plasticity, and degeneration. However, due to technical limitations MWI may report biased values of the true myelin concentration. In order to improve the accuracy of $MWF$ estimates, we acquired and jointly analyzed ME-GE and ME-SE imaging data. MWI requires the estimation of the respective relaxation spectra, which is a highly ill-posed inverse problem. Strong regularization is typically required to enforce a stable solution, which usually results in biased $MWF$ estimates. We hypothesized that jointly inverting both datasets reduces the ill-posedness of the inverse problem, potentially relaxing the need for strong regularization. For this, it was implicitly assumed that ME-GE and ME-SE provide independent measures of $MWF$. Under this assumption, we formulated an objective function with data consistency terms for ME-GE and ME-SE data. The forward operators to compute the respective signals were constructed through parameterized two-pool Gaussian spectra describing the MW and AEW components. For ME-GE and ME-SE signals, they are given by the ${T}_{2}^{*}$ and the ${T}_{2}$ voxel-distribution, respectively. Under the stated assumption, both distributions share the same $MWF$ which therefore can be jointly inverted from both datasets.

Our results suggest that the joint inversion approach mitigates the ill-posed nature of the inversion problem inherent in MWI. The underdetermined inverse problem can lead to ambiguity and instability in the reconstruction process when relying on a single dataset. By combining information from both sequences, our method introduces additional constraints that inherently help to regularize the inversion process, thereby improving the stability and accuracy of the estimated $MWF$.

The simulation results presented in this study demonstrate the superiority of the proposed joint inversion approach over conventional methods that rely on a single dataset for MWI. The main advantage of the simulation is the knowledge of the ground truth (completely defined by the known ${T}_{2}$ and ${T}_{2}^{*}$ model), which enables to determine and compare the accuracy of the different inversion methods. For the single ${T}_{2}$ and ${T}_{2}^{*}$ inversions, we observed under- and overestimation for a wide range of the true $MWF$ values. Instead, the $MWF$ joint inversion estimates were closer to the ground truth in almost all cases. These results were obtained for SNR = 150 of the respective first echo of the synthetic data. In simulations with much lower SNR, all three estimates, also the join inversion, significantly deviate from the true $MWF$ values. If the SNR is increased beyond 150, the advantage of the joint inversion remains but gets smaller, since also the bias of the individual inversions decreases. Thus, the improvement in accuracy seems to be particularly noteworthy when considering the challenges posed by realistic SNR.

The superiority of the joint inversion approach is further corroborated by the results obtained from in vivo data. Here, the ground truth is not known and biases due to incorrect model assumptions may enter as well (cf. subsection Limitations below). In contrast, the joint inversion produces sharper MW fraction maps which highlight its ability to provide enhanced spatial resolution and tissue contrast compared to the conventional approaches. The improved level of detail is a result of leveraging additional information from a second dataset during the joint inversion process. The two datasets have different sensitivities and resolution characteristics. This complementary information reduces the influence of regularization although the same regularization method and strength were applied to single and joint inversions. The joint inversion problem is less ill-posed and less impacted by regularization, which lead to more detailed images. The improved results of the joint inversion are not related to enhanced SNR from the bigger dataset, as it was compared to the single inversions of two repeated scans, which resulted in approximately the same amount of data and scan time. Indeed, the results from single inversions on single datasets (not shown), are very similar to those from the averaged scans. This might be attributed to the high SNR already present in the single acquisitions. The joint inversion is better constrained since it inverts simultaneously multiple types of data, which help to reduce the solution space in comparison with the single inversions. The joint inversion can also average out the noise from the single datasets, leading to more reliable results. Moreover, ${T}_{2}$ and ${T}_{2}^{*}$ inversions seem to solve the minimization problems differently: the $MW{F}_{{T}_{2}}$ maps are less noisy while the $MW{F}_{{T}_{2}^{*}}$ maps are less blurred (in case of a complex signal model, cf. Figure 5). Joint inversion leverages the strengths of both data types, leading to higher resolution models. Noteworthy, the group statistics in WM ROIs (cf. Table 2) show that the joint inversion $MWF$ is mostly larger than the estimates of the single inversions. However, due to the lack of a ground-truth, it remains to be shown if the in vivo $MWF$ estimates are more accurate than those obtained from the single inversions. Although this observation is supported by the simulation results, it cannot be generalized to the case of in vivo data (cf. limitations below). Nevertheless, the main advantage of the joint inversion approach is the obvious improvement in resolution as compared to the results based on a single dataset. Table 2 also shows that the different fits of the MW and AEW relaxation times are in good agreement in most ROIs. This indicates that the joint inversion consistently found the same local optima for the respective distributions (${T}_{2}$ and ${T}_{2}^{*}$) as the single inversions. Noteworthy are also the small group standard deviations of all estimates and ROIs, indicating that all inversion approaches provide stable cross-subject results in healthy volunteers. However, this observation needs to be confirmed using a larger sample, as only three subjects were included in this proof-of-concept study.

Several limitations warrant consideration. First, the simulation results have to be interpreted with care since the data are modeled with the same algorithm as used for inversion. This scenario is called *inverse crime* in geophysics: during the iterative fitting process, the data consistency terms will produce perfect gradient estimation and therefore facilitating convergence during the inversion. In real data, the residuals in addition contain deficiencies in the modeling algorithm that leads to gradient errors too. Thus, for instance, it would be not useful to compare the performance of conventional NNLS inversion with parameterized inversion while using the same parametric model for generating synthetic data. In that case the data would perfectly fit to one approach but not to the other, and the results would be misleading. Therefore, NNLS was not considered in the simulations. For the in vivo data we also performed conventional NNLS inversion individually on ME-GE and ME-SE data. The results (not shown) were similar but slightly worse as compared to the parameterized single inversions, as expected [6]. Another limitation of the current approach is that ${B}_{0}$ field inhomogeneities were not taken into account. Due to the high readout bandwidth of the ME-GE sequence image distortions are minimal, yet the multi-exponential ${T}_{2}^{*}$ decay model might be invalid in regions with large $\Delta {B}_{0}$ [34]. Furthermore, phase images contain multiple phase wraps in these regions, which results in unstable $MWF$ fits for both, single inversion ME-GE and joint inversion (cf. $MWF$ maps in the supplementary material). Improved phase preprocessing may help to overcome these problems. However, since we mainly focused on $MWF$ estimates in WM, we assume that susceptibility-induced field inhomogeneities have minor influence on the presented results. The proposed inversion method, especially the proposed initial model and regularization parameters, may be sensitive to variations in acquisition parameters and pathology-dependent tissue properties, necessitating further validation across different imaging protocols and patient populations. Finally, the results were obtained on high SNR data resulting from rather lengthy acquisitions (20 min), as good data quality was seen as a requirement for this proof-of-concept study. The impact of reduced SNR in accelerated acquisitions needs to be investigated in future, aiming at faster protocols suitable for patient studies.

In conclusion, this study demonstrates the effectiveness of a novel joint inversion approach for MWI, which integrates information from multi-echo spin-echo and gradient-echo imaging sequences to improve the accuracy and reliability of MW quantification. The observed improvements in accuracy and spatial resolution, coupled with the potential clinical implications, underscore the significance of our proposed method in advancing neuroimaging research and clinical practice. Future work will focus on further refining the proposed approach and validating its utility in clinical studies, e.g., multiple sclerosis (MS) patients.

## Supplementary Information

Below is the link to the electronic supplementary material.

Supplementary file1 (DOCX 6486 KB)
