# Supplementary material for: Myelin water fraction mapping with joint inversion of gradient-echo and spin-echo data
Source: MAGMA. 2025 Mar 7;38(2):317–32. doi: 10.1007/s10334-025-01235-5 (PMC11914316; doi:10.1007/s10334-025-01235-5)
Supplement: Supplementary file 1 — Supplementary file1 (DOCX 6486 KB) [file 10334_2025_1235_MOESM1_ESM.docx]

## Supporting Information


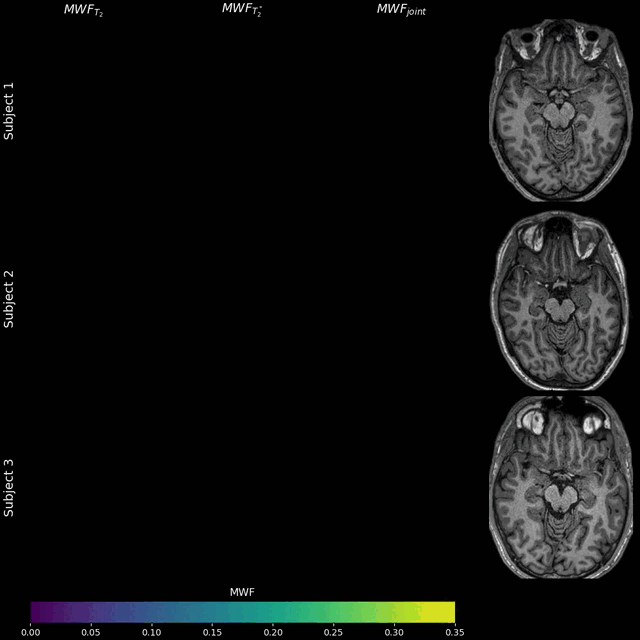


S1: Animated gif video, showing all slices of the MWF results corresponding to Fig. 5.


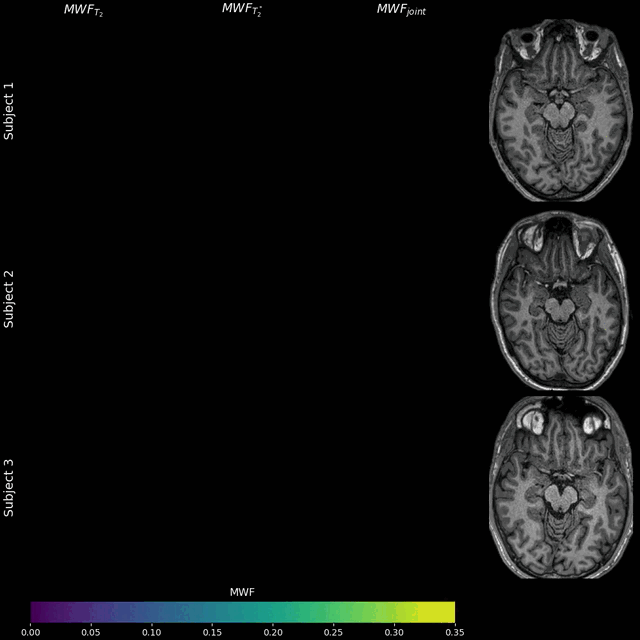


S2: Animated gif video, showing all slices of the MWF results corresponding to Fig. 6.


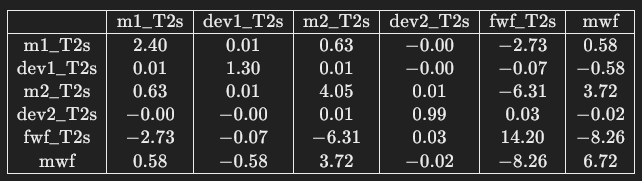


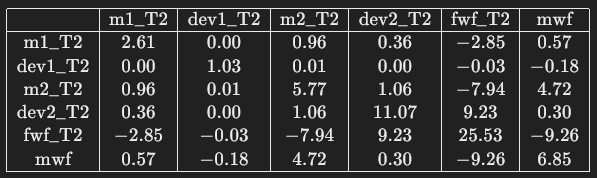


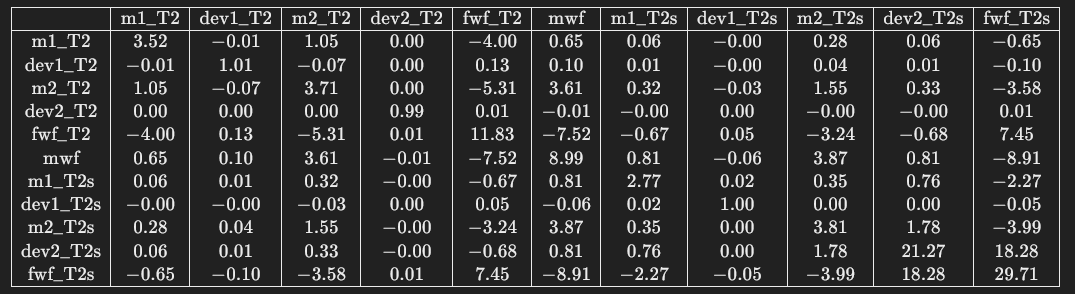


S4: Correlation Analysis of the inversion parameters. The tables show the covariance matrix of the model parameters for all three inversions. **Top**: single T2 inversion, **Middle**: single T2* inversion, **Bottom**: joint inversion. Diagonal elements give the variance (uncertainty) of individual parameters, and off-diagonal elements represent the correlation between pairs of parameters. Ideally, off-diagonal elements are close to 0 when they are not correlated with each other. Relation to the math symbols in the main document: m1_T2 = $\mu_{1,T_{2}}$, dev1_T2=$\sigma_{1,T_{2}}$, m1_T2 = $\mu_{2,T_{2}}$, dev2_T2=$\sigma_{2,T_{2}}$, fwf_T2=$I_{2,T_{2}}$ mwf = $MWF,$ m1_T2s = $\mu_{1,T_{2}^{*}}$, dev1_T2s=$\sigma_{2,T_{2}}$, m1_T2s= $\mu_{2,T_{2}^{*}}$, dev2_T2s=$\sigma_{2,T_{2}^{*}}$, fwf_T2s=$I_{2,T_{2}^{*}}$.
